# Metabolic profile and gene expression pattern of cytokines and antioxidants markers during different physiological stages in Barki ewes

**DOI:** 10.1186/s12917-024-04018-7

**Published:** 2024-05-17

**Authors:** Ahmed Adel El-Sayed, Ahmed M. Sallam, Ibrahim Abou-Soliman

**Affiliations:** 1 Department of Animal Health and Poultry, Animal and Poultry Production Division, Desert Research Center (DRC), Mataryia, Cairo Egypt; 2 Department of Animal and Poultry Breeding, Animal and Poultry Production Division, Desert Research Center (DRC), Mataryia, Cairo Egypt

**Keywords:** Barki sheep, Acute phase proteins, Oxidative stress, Gene expression, Physiological stages

## Abstract

**Background:**

In livestock, identifying the physiological and reproductive stages is valuable in guiding management decisions related to nutrition, veterinary procedures, and breeding programs. To achieve this goal, a cohort of Barki ewes in this research underwent observation across three pivotal physiological conditions: pre-pregnancy, late pregnancy, and early lactation. Blood samples were collected to investigate the changes in serum metabolic profile as well as gene expression pattern of cytokines and antioxidants markers during these stages.

**Results:**

Our results showed that during late pregnancy, there was a significant (*P* < 0.05) increase in red blood cells **(11.9 ± 0.5 10**^**12**^**/L)**, hemoglobin (10.8 ± 0.4 g/dl) and neutrophils count (7 ± 0.1 10^9^/L) with significant decrease (*P* < 0.05) of total white blood cell count (9.1 ± 0.05 10^9^/L). The packed cell volume (%) and monocyte count showed a significant (*P* < 0.05) decrease during both late pregnancy and early lactation stages. The serum concentrations of glucose, cholesterol, GSH, GPx, SOD and catalase displayed significant (*P* < 0.05) decrease during late pregnancy and early-lactation. Notably, during late pregnancy, there was a significant (*P* < 0.05) increase in the serum concentrations of albumin, globulin, urea, IGF-1, and malondialdehyde with significant decrease (*P* < 0.05) of total protein (4.9 ± 0.08 g/dl). Additionally, during early lactation, there was a significant (*P* < 0.05) increase in the serum levels of non-esterified fatty acids, triiodothyronine (T3), and thyroxin (T4). The gene expression profiles of cytokines (*IL-4, IL-6, IL-8*, and *NFKB*) were decreased in the ewes during late pregnancy compared to pre-pregnant and early lactation stages. In addition, the expression profile of antioxidant genes (*SOD, CAT, GPX*, and *Nrf2*) was significantly upsurged in the non-pregnant ewes compared to late pregnancy and early lactation ones.

**Conclusions:**

The results concluded that different physiological status significantly affects the blood metabolic profile and gene expression pattern in Barki sheep. Our findings can be helpful in monitoring animal health and applying in breeding programs of Barki sheep under harsh environmental conditions.

## Introduction

Barki sheep breed, named after the Libyan province Barka, is indigenous to the north-western coastal zone of Egypt, and holds an essential part of the livelihood of the local population in the region [1]. It spans a wide geographical area that extends from eastern Libya to the western region of Alexandria in Egypt. It plays a dominant role in this region exhibiting a high potential for adaptation to the harsh climate conditions, limited forages, and heat stress [2].

The nutritional requirements of an animal are determined by its physiological stage [3]. Among these stages, the periparturient period, encompassing the end of pregnancy and the onset of lactation holds particular importance. This critical stage is characterized by substantial changes in feed intake, the delivery process, and beginning of lactation [4]. Additionally, the transition from pregnancy to lactation involves not only dietary adjustments but also hormonal shifts and homeostatic modifications to supply nutrients for fetal development and lactogenesis [5]. Consequently, a common challenge during this period is the occurrence of negative energy balance due to a shortage in feed intake and an increase in nutritional requirements, potentially leading to immunosuppression consequences [6]. Furthermore, metabolic disorders such as hypocalcemia, hypomagnesemia, fatty liver syndrome, and pregnancy toxemia may manifest during these stages [7, 8]. Besides, the mobilization of body reserves alters the concentrations of the serum non-esterified fatty acids (NEFA) and beta-hydroxybutyric acid (β-HBA) [9]. Similarly, alterations in blood metabolites including insulin, glucose, protein, cholesterol, triglyceride and creatinine have been reported [10]. Identifying these metabolic changes in a timely manner is crucial in early detection of disorders, providing useful information to farmers and breeding companies to avoid losses in their flocks [11].

The acute-phase proteins (APPs) produced in liver were used as an assessment tool for different disorders in animals [12]. The level of APPs is influenced by various pathogenic (e.g., viral and non-infectious disorders), physiological (e.g., nutrition, pregnancy and lactation) and environmental factors [13]. So that, identifying the variability in APPs levels at different physiological conditions may be an indicator of animal health and reproduction [14]. Oxidative stress commonly occurs when there is an excess in oxidants, such as free radicals or reactive oxygen species coupled with a decline in antioxidants [15]. Nevertheless, animal bodies have mechanisms to regulate the overproduction of free radicals through protective mechanisms [16]. The antioxidant enzymes, such as catalases, play important role in these mechanisms [17].

Understanding the animal response to stress under different physiological conditions may provide new insights into their genetic potentials to adaptation. Recently, genetic selection for animal adaptation to harsh conditions has been a compelling research topic. Identifying variations in the expression levels of regulatory genes enhances our understanding of the adaptation process [18]. These genes could be integrated in selection programs that aim at developing resilient animals capable of producing under stress without significant losses [19]. Limited information is available regarding alterations in APPs, metabolic profiles, oxidative stress markers and the pattern of gene expression of inflammatory markers at important physiological stages in Barki ewes. Therefore, the objective of the current study is to provide a concise overview of these changes before pregnancy, during late pregnancy and at early lactation stages in Barki ewes.

## Materials and methods

### Animals and study design

A total of 40 seemingly healthy non pregnant non lactating Barki ewes with an average of 4–6 years (mean ± SD: 4.9 ± 0.7) and a range of bodyweight 28–45 kg (mean ± SD: 38.5 ± 4.9) were used in this study. The experiment was carried out in Mariut Research Station, Desert Research Center, El-Amryea, Alexandria, Egypt during the period between August 2022 and June 2023. In October 2022, natural mating was introduced to all investigated ewes. Samsung Medison SONOACE R3 ultrasound equipment, South Korea, confirmed that only thirty ewes were pregnant. These ewes were then retained for further investigations during the physiological periods of interest. The ewes were housed in semi-open, shaded yards and received the same diet of ad libitum feeding on Egyptian clover hay and concentrated mixture with freely available minerals blocks and water resources.

### Blood sampling

Three steps were taken in the blood sample process. The study began with a blood sample from all examined ewes (*n* = 40) that were not pregnant or lactating, which was referred to as group 1. The next step involved the last two to four weeks of confirmed pregnancy for ewes which was referred to as group 2 (late pregnant ewes, *n* = 30), and the third step involved the first one to two weeks following delivery, which was referred to as group 3 (early lactating ewes, *n* = 30). All ewes under this study **were healthy and exhibited regular estrous cycle and free from any reproductive disorders.** All ewes were almost at the same stage of pregnancy (30 ± 5 days) at the time of sampling, conceived and gave birth normally without any signs of complications. All lambs **were healthy and suckling their dams’** milk until weaning at three months of lambs, age. Blood samples were collected via jugular vein into plain tubes without anticoagulants and tubes containing EDTA. The complete blood count (CBC) was determined from the whole blood of each sample **using an automatic blood cell counter (Exigoeos veterinary Hematology system, Boule Medical AB, Sweden).** Only clear sera and plasma were collected, aliquoted and kept frozen at -20^C^ for the subsequent analyses.

### Biochemical analysis

Serum biochemical concentrations were measured using commercial test kits in accordance with standard protocols. The serum amyliod A (SAA) and plasma fibrinogen (Fb) concentrations were assessed using IBL International Crop (Canada)® ELISA kits, and haptoglobin (Hp) by Eagle Biosciences (Columbia) ELISA kits. The total protein, albumin, glucose, cholesterol, urea, and creatinine were determined by kits from Gamma Trade Company, Egypt. The insulin levels were measured by kits from NeoBiolab, Cambridge, USA. For T3 and T4 commercial test, Malondialdehyde (MDA), catalase (CAT), reduced glutathione (GSH), super oxide dismutase (SOD), Glutathione peroxidase (GPx) and alkaline phosphatase (ALP) were provided by Bio diagnostic, Egypt. Betahydroxybutyrate (BHBA) provided by Cayman chemical Company, USA. NEFA kits from FUJIFILM Wako Chemicals, USA. The IGF1 kits from Wuhan Fine Biotech. Co, China. Total bilirubin from Atlas Medical, Germany. Globulin was calculated by subtracting albumin values from total serum protein.

### RNA extraction and quantitative real time PCR

For each whole blood sample, the total RNA was extracted using Trizol reagent following the manufacturer’s protocol (RNeasy Mini Ki, Catalogue no.74,104). Quality and quantity of the extracted RNA was assessed using NanoDrop®ND-1000 Spectrophotometer. Subsequently, cDNA was synthesized for each sample in accordance with the manufacture protocol (Thermo Fisher, Catalog no, EP0441). To determine the relative mRNA levels of the target genes, RT-PCR was conducted using SYBR Green PCR Master Mix (Quantitect SYBR green PCR kit, Catalog no, 204,141). Primer sequences were designed based on *Ovis Aries* reference genome (Table 1).

**Table 1:** Oligonucleotide primers sequence, annealing temperature and PCR product size of the studied genes

Gene	Oligonucleotide sequence (5′-3′)	Accession number	AT (^0^C)	Amplicon size (bp)
***IL-4***	F- GTACCAGCCACTTCGTCCA- R-GTGGCTCCTGTAGATACGCC	NM_001009313.3	58	200
***IL-6***	F-TGCAGTCCTCAAACGAGTGG R- CCGCAGCTACTTCATCCGA	NM_001009392.1	58	110
***IL-8***	F- GACCCCAAGGAAAAGTGGG R-CCACACAGTACTCAAGGCACT	NM_001009401.2	62	183
***NFKB***	F-GCCTTTGGGGACTTCTCTCC- R- GCAGGAACACGGTTACAGGA	AF283892.1	58	109
***SOD1***	F-TGATCATGGGTTCCACGTCC- R-CACATTGCCCAGGTCTCCAA-	NM_001145185.2	60	139
***CAT***	F-CAGTAGGAGACAAACTCAAT R-ACGACTCTCTCAGGAATTCTC	GQ204786.1	62	121
***GPX1***	F-CGAGGAGATCCTGAATTGCCTGA R-ACCTCGCACTTTTCGAAGAGC	JF728302.1	60	95
***Nrf2***	F-GCAGTTCACTCAGTGCCATC- R-TACCTCTCGACTTACCCCGA-	XM_012132956.4	58	249
***GAPDH***	F- TGACCCCTTCATTGACCTTC- R-GATCTCGCTCCTGGAAGAG	NM-001034034	62	143

IL-4 = Interleukin-4; IL-6 = Interleukin-6; IL-8 = Interleukin-8; NFKB = Nuclear factor kappa B; SOD1 = Superoxide dismutase 1; CAT = Catalase; GPX1 = Glutathione peroxidase 1; Nrf2 = Nuclear factor-erythroid factor 2-related factor and GAPDH = Glyceraldehyde-3-Phosphate Dehydrogenase. AT = annealing temperature, bp = base pair

For each sample, the 25 µl total reaction volume consisted of a mixture of 3 µl of total RNA, 4 µl of 5x Trans Amp buffer, 0.25 µl of reverse transcriptase, 0.5 µl of each primer, 12.5 µl of 2x Quantitect SYBR green PCR master mix and 8.25 µl of RNase free water. The thermal cycler PCR was used to amplify the target sequence of the cytokines (*IL-4, IL-6, IL8*, and *NFKB*) and antioxidant (*SOD1, CAT, GPX1*, and *Nrf2*) genes following this PCR program: Initial reverse transcription at 50 °C for 30 min, primary denaturation at 94 °C for 10 min followed by 40 cycles of 94 °C for 15 s, annealing at temperatures specified in Table 2, and extension at 72 °C for 30 s. After the amplification, a melting curve analysis was conducted to confirm the specificity of each PCR product. The *ß. actin* housekeeping gene was used as a constitutive control. The relative expression of each gene for each sample in comparison with *ß. actin* gene was determined and calculated using the 2^−ΔΔCt^ method [20].

**Table 2:** Hematological parameters in Barki ewes during different physiological phases (mean ± SE)

Parameters	Pre-pregnant – non lactating	Late pregnant	Early lactating	*P* value
**RBC (× 10** ^**12**^ **/L)**	9.6 ± 0.08^a^	11.9 ± 0.5 ^b^	8.9 ± 0.3 ^a^	0.004
**Hb (g/dl)**	9.3 ± 0.3 ^a^	10.8 ± 0.4 ^b^	9.2 ± 0.1 ^a^	0.02
**PCV%**	58.6 ± 2.6 ^a^	51.6 ± 1.7 ^b^	45.3 ± 3.7 ^b^	0.04
**MCV (fL)**	53.1. ± 0.8	51.1 ± 0.4	39.5 ± 1.2	0.28
**MCH (pg)**	10.5 ± 0.1	10.3 ± 0.05	10.3 ± 0.05	0.1
**MCHC (g/dl)**	19.7 ± 0.1	20.2 ± 0.2	19.8 ± 0.1	0.18
**WBC(×10** ^**9**^ **/L)**	10.7 ± 0.2 ^a^	9.1 ± 0.05 ^b^	12.5 ± 0.2 ^a^	0.001
**Neutrophil (× 10** ^**9**^ **/L)**	5.2 ± 0.2 ^a^	7 ± 0.1 ^b^	9.7 ± 0.2 ^b^	0.001
**Monocyte (×10** ^**9**^ **/L)**	0.3 ± 0.008 ^a^	0.1 ± 0.006 ^b^	0.2 ± 0.01 ^b^	0.001
**Lymphocyte (×10** ^**9**^ **/L)**	3.2 ± 0.05	3.2 ± 0.1	3.1 ± 0.06	0.9

RBC: Red blood cells; Hb: Hemoglobin; PCV: Packed cell volume; MCV: Mean corpuscular volume; MCH: Mean corpuscular hemoglobin; MCHC: Mean corpuscular hemoglobin concentration; WBC: White blood cells. a, b, c Different superscripts indicate significant differences (*P* < 0.05) between the various study group

### Statistical analysis

Statistical analyses were carried out using a statistical software program (SPSS, ver.20, Inc., Chicago, USA). Descriptive statistics were performed for all parameters. A repeated measure ANOVA was used to test the effect of different physiological status on metabolic and gene expression pattern of cytokines and antioxidants markers. Results were considered statistically significant at *P* ˂ 0.05.

## Results

### Haemato-biochemical parameters

An overview of serial measurements of hematological and serum biochemical profile in Barki ewes at different physiological states is illustrated in Tables 2 and 3, respectively. The investigated ewes did not show any clinical alterations throughout the study period and remain clinically healthy.

**Table 3:** Biochemical parameters in Barki ewes during different physiological phases (mean ± SE)

Parameters	Pre-pregnant – non lactating	Late pregnant	Early lactating	*P* value
**Glucose (mg/dl)**	87.6 ± 1.4^a^	73.3 ± 1.1 ^b^	71.1 ± 0.4 ^b^	0.001
**Cholesterol (mg/dl)**	76.2 ± 0.3^a^	47.7 ± 0.5 ^b^	48 ± 0.5 ^b^	0.001
**Total protein (g/dl)**	6.7 ± 0.1^a^	4.9 ± 0.08 ^b^	6.7 ± 0.05 ^a^	0.001
**Albumin (g/dl)**	3.4 ± 0.1^a^	4.8 ± 0.05 ^b^	3.3 ± 0.1 ^a^	0.001
**Globulin (g/dl)**	1.5 ± 0.05^a^	2.4 ± 0.1 ^b^	1.4 ± 0.05 ^a^	0.001
**Urea (mg/dl)**	31.8 ± 0.7^a^	45.8 ± 1.6 ^b^	30.6 ± 0.8 ^a^	0.001
**Creatinine (mg/dl)**	1.1 ± 0.05 ^a^	1.1 ± 0.02 ^a^	1.1 ± 0.01 ^a^	0.3
**Total bilirubin (mg/dl)**	0.26 ± 0.08^a^	0.26 ± 0.01 ^a^	0.26 ± 0.01^a^	0.9
**ALP (U/L)**	185 ± 5.7^b^	96 ± 4 ^a^	99.3 ± 3.1 ^a^	0.001
**NEFA (mmol/l)**	0.3 ± 0.005^a^	0.3 ± 0.01 ^a^	1.8 ± 0.05 ^b^	0.001
**BHBA (mmol/l)**	0.4 ± 0.02^a^	0.96 ± 0.03 ^b^	0.94 ± 0.03 ^b^	0.001
**Insulin (µIU/ml)**	4.9 ± 0.05^a^	5 ± 0.08 ^a^	4.8 ± 0.2 ^a^	0.7
**T3 (pg/ml)**	98 ± 2.3^a^	106.3 ± 3.1 ^a^	165.6 ± 2 ^b^	0.001
**T4 (pg/ml)**	5.7 ± 0.07^a^	5.4 ± 0.1 ^a^	7.5 ± 0.1 ^b^	0.001
**IGF1 (ng/ml)**	3.6 ± 0.1^a^	6.9 ± 0.05 ^b^	3.6 ± 0.1^a^	0.001
**GSH (mg/dl)**	37.3 ± 1.7^a^	19.1 ± 0.6 ^b^	19.6 ± 0.3 ^b^	0.001
**GPx (U/gHb)**	57.3 ± 1.4^a^	29 ± 0.5 ^b^	27.3 ± 0.8 ^b^	0.001
**SOD (U/ml)**	63.8 ± 0.9^a^	31 ± 0.5 ^b^	32 ± 0.5 ^b^	0.001
**Catalase (U/l)**	50 ± 0.5^a^	23.1 ± 0.4 ^b^	24 ± 0.5 ^b^	0.001
**MDA (nmol/ml)**	7.2 ± 0.2^a^	14.5 ± 0.2 ^b^	7.9 ± 0.3 ^a^	0.001
**Hp (ng/ml)**	40.3 ± 2^a^	52 ± 1.1 ^b^	65.6 ± 2.9 ^c^	0.001
**SAA (mg/l)**	4.6 ± 0.05^a^	4.7 ± 0.07 ^a^	4.7 ± 0.1 ^a^	0.3
**Fb (g/L)**	4.6 ± 0.05^a^	4.4 ± 0.08 ^a^	4.5 ± 0.1 ^a^	0.7

ALP: Alkaline phosphatase; NEFA: Non-Esterified Fatty Acids; BHBA: Beta-hydroxy-butyric acid; T3: Triiodothyronine; T4: Thyroxine; IGF1; Insulin-like Growth Factor 1, GSH: Glutathione reduced; GPx: Glutathione peroxidase; SOD; Superoxide dismutases MDA: Malondialdhyde; Hp: Haptoglobin. SAA; Serum amyloid A. Fb; Fibrinogen. a, b, c Different superscripts indicate significant differences (*P* < 0.05) between the various study groups

Table 2 summarizes the hematological variables in the examined ewes. The TEC and Hb showed a significant (*P* < 0.05) increase in the ewes at late pregnancy compared to early-lactation and pre-pregnancy stages coupled with significant (*P* < 0.05) decrease in PCV (%) values at late pregnancy and early-lactation compared to the pre-pregnancy stage. Total WBCs count decreased significantly (*P* < 0.05) at late pregnancy compared to the pre-pregnancy and early lactating stage. The differential leucocytic count showed significant (*P* < 0.05) increase in neutrophil and significant (*P* < 0.05) decrease in monocyte count in ewes at late-pregnancy and early lactation stages compared to the pre-pregnancy stage. All other hematologic variables and the related indices were not significantly (*P* > 0.05) differed among the tested time points.

Biochemically, there was a significant (*P* < 0.05) decrease of serum concentrations of glucose, cholesterol, GSH, GPx, SOD and catalase and significant (*P* < 0.05) increase of BHBA and Hp concentration in the ewes at late pregnancy and early-lactation compared to the pre-pregnant stage. In addition, there was significant (*P* < 0.05) increase of serum level of albumin, globulin, urea, IGF-1 and MDA with significant (*P* > 0.05) decrease of TP level in the ewes at late-pregnancy compared to early-lactation and pre-pregnancy stages (Table 3). Moreover, there was a significant (*P* < 0.05) increase of serum level of ALP activity in ewes at the pre-pregnancy compared to late pregnancy and early lactation stage while the serum level of NEFA increased significantly (*P* < 0.05) in the ewes at early lactation compared to the other stages. On the other hand, there was a significant (*P* < 0.05) increase of serum level of T3 and T4 in the ewes at early lactation compared to the pre-pregnancy and late pregnancy. No significant differences in serum concentrations of insulin, creatinine, total bilirubin, SAA and fibrinogen at the study stages were observed (Table 3).

### Gene expression of cytokines and antioxidant markers

**Fig. 1:**
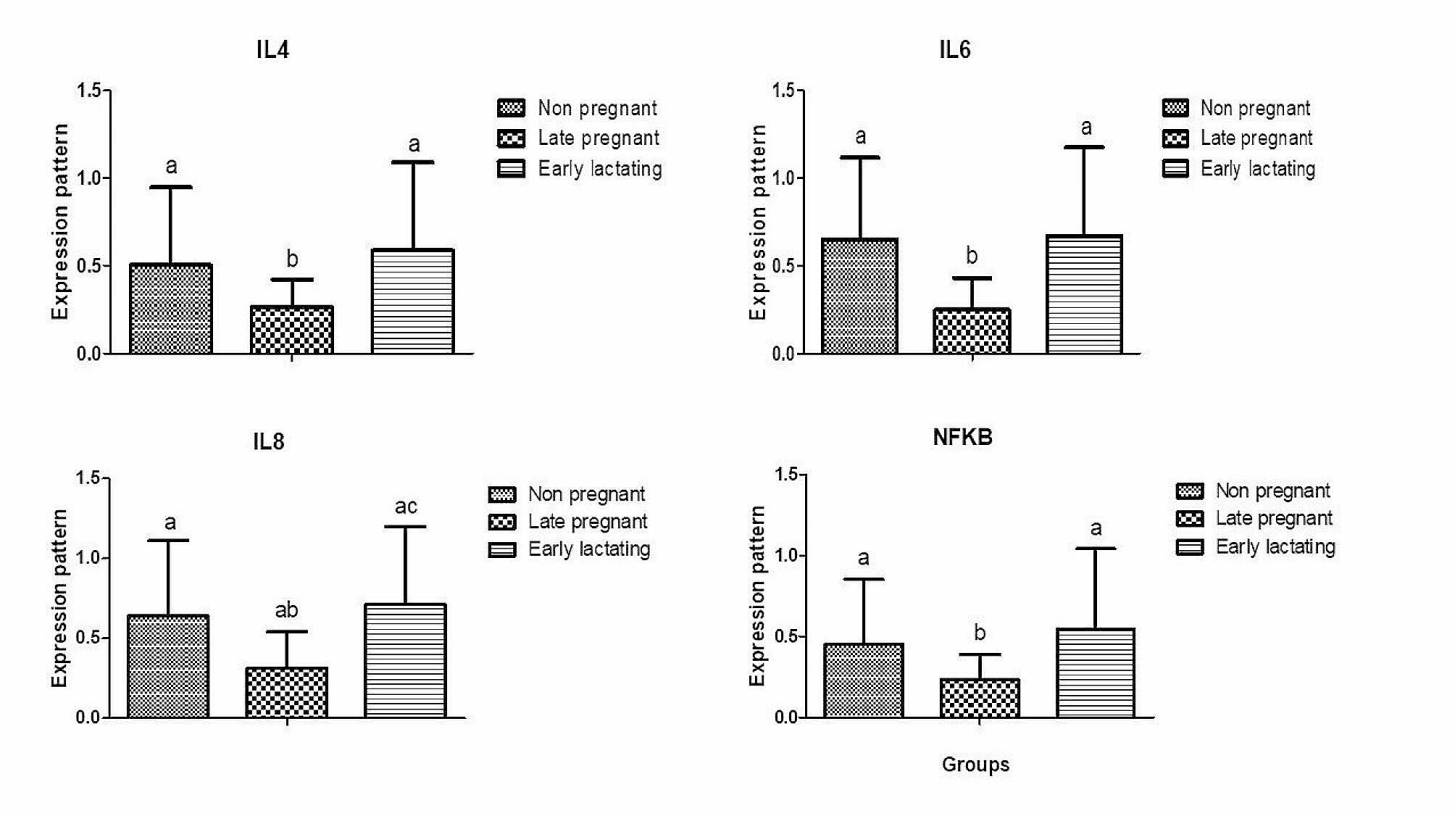
Gene expression profile of immune markers (IL4, IL6, IL8 and NFKB) in non-pregnant, late pregnant, and early pregnant ewes. **a**, **b**, **c** Different superscripts indicate significant differences (*P* < 0.05) between the various study groups

**Fig. 2:**
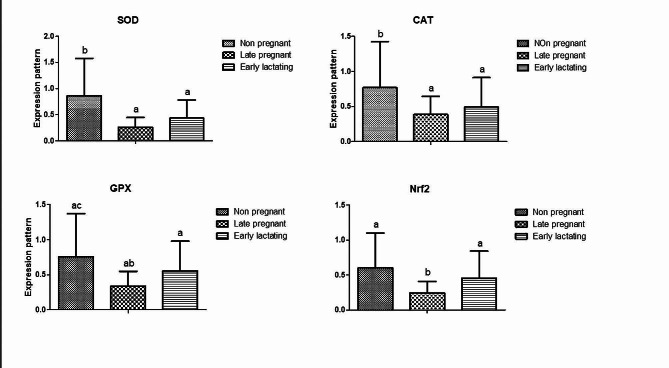
Gene expression profile of oxiditive stress markers (SOD, CAT, GPX and Nrf2) in non-pregnant, late pregnant, and early pregnant ewes. **a**, **b**, **c** Different superscripts indicate significant differences (*P* < 0.05) between the various study groups

The gene expression profiles in pre-pregnant, late pregnant and early lactation Barki ewes of cytokines and antioxidant markers are presented in Figs. 1 and 2, respectively. Results showed that the expression profile of cytokine genes (*IL-4, IL-6, IL-8*, and *NFKB*) in the ewes at late pregnancy was diminshed compared to pre-pregnancy and early lactation stages. Furthermore, the expression profile of antioxidant genes (*SOD, CAT, GPX*, and *Nrf2*) was significantly increased in the pre-pregant ewes compared to late pregnancy and early lactation stages. For *SOD* gene, the highest potential quantity of mRNA was found in the ewes at pre-pregnancy stage (1.57 ± 0.15), whereas the lowest amount was found in *NFKB* (0.85 ± 0.05). The *CAT* had the highest expression level (0.64 ± 0.12) among all genes analyzed in the ewes at late pregnancy stage, while *NFKB* had the lowest level (0.38 ± 0.07). During early lactation, the *NFKB* gene had the lowest expression level (0.76 ± 0.11), while the *IL-8* gene had the highest expression level (1.19 ± 0.23).

### Correlation between gene expression and serum profiles of cytokines and antioxidant markers during different physiological stages

In non-pregnant non lactating group, the serum levels of SAA were positively correlated with mRNA levels of IL4 (*r* = 1 and *p* = 0.009). In late pregnant group, the serum levels of Hp were positively correlated with mRNA levels of GPX and Nrf2 gene (*r* = 0.999 and *p* = 0.02, *r* = 1and *p* = 0.011, respectively). In early lactating group, the serum levels of GPX were positively correlated with mRNA levels of CAT and GPX gene (*r* = 1 and *p* = 0.01, *r* = 0.997 and *p* = 0.04, respectively), serum levels of Hp were negatively correlated with mRNA levels of CAT (*r*= -1 and *p* = 0.01), serum levels of SAA were negatively correlated with mRNA levels of SOD gene (*r*= -0.998 and *p* = 0.04) and serum levels of Fb were positively correlated with mRNA levels of IL4 gene (*r* = 1 and *p* = 0.01) .

## Discussion

This study provided the first comprehensive overview of metabolic and gene expression patterns of cytokines and antioxidant markers at critical physiological stages in Barki ewes.

There was significant increase in TEC and Hb levels observed in our study during late pregnancy in ewes compared to early lactation and the pre-pregnant stage, with significant decrease in packed cell volume (PCV) values at late pregnancy and early lactation compared to the pre-pregnant stage. These results were consistancy with findings in other studies on sheep [21] and goats [22]. However, no changes in these hematological parameters were reported in other sheep, cattle, and camel breeds [39]. This elevation in TEC and Hb levels may be due to higher oxygen demand and increased metabolic rate during pregnancy, or it could be linked to augmentation in RBC volume during pregnancy. The decline in PCV (%) during late pregnancy and early lactation may be a result of the increased Hb levels in RBCs plasma or the vascular system, leading to enhanced water mobilization in the mammary glands [23].

In the examined sheep, the total WBC count significantly decreased in late pregnancy as compared to pre-pregnancy and early lactation phases. Similar result was also reported in **Ossimi sheep breeds** [24]. Similarly, the findings in the lactating buffaloes compared to the pregnant [42] were inconsistent with the increase in total WBC count in Barki ewes during lactation compared to late pregnancy. In contrast [41], observed an increase in the leukocyte count in the pregnant Sahel goats, attributing it to increased bone marrow activity and stress during pregnancy. These findings suggest that uterine involution may be the reason for the low total WBC count throughout pregnancy, with an increase during parturition and early lactation stages [25].

The differential **leukocytic** count displayed a significant increase in neutrophil count with significant decrease in monocyte values at late pregnancy and early lactation compared to pre-pregnancy stage. These results are consistent with those reported in **Ossimi sheep** [39] and camels [42]. The negligible alterations in lymphocyte count **in the current study align** with findings in Ossimi sheep and goats [37, 39]. The decreased values of monocytes during early lactation also agree with observations in Ossimi sheep [39]. This could be speculated as pregnancy-related stress stimulating the anterior pituitary gland to secrete ACTH, prompting the glucocorticoids production from adrenal cortex. These components are involved in mobilization of neutrophils from the body pool into the peripheral circulation [26].

In comparison to the pre-pregnant period, Barki ewes’ serum glucose concentrations significantly decreased during the late pregnancy and early lactation stages. However, there was significant reduction in TP concentrations during late pregnancy as compared with other physiological stages. These results were consistent with those reported in sheep [39], goats [46] and camels [47]. Conversely [48], reported higher serum glucose and total protein concentrations at early lactating in the African dwarf does compared to other physiological states. Non-significant differences in serum TP in cattle during the third trimester of pregnancy and early lactation was reported as well [49]. The significant decrease of glucose levels during late pregnancy and early lactation stages in the current study may be attributed to the mobilization of maternal glucose to fetal blood circulation, which is essential for fetal development [27], as well as the energy loss during milk synthesis [28]. Similarly, the significant reduction in TP concentrations during late pregnancy observed in the current study could be due to the high requirements for fetal growth during this period [29]. Additionally, the protein utilized for colostrum formation may contribute to a decline in protein concentration in early nursing ewes [30].

The considerable drop in serum cholesterol levels during late pregnancy and early lactation stages in ewes, as observed in the current study, is consistent with findings in sheep [31] and goats [32]. However, higher serum cholesterol levels were reported during late pregnancy and early lactation in sheep [36] and goats [37]. Additionally [48], demonstrated non-significant variation in cholesterol levels across several physiological statuses in goats. This decline could be attributed to increased cholesterol uptake by tissues involved in milk synthesis, coupled with the role of total cholesterol in ovarian steroidogenesis.

When compared to the early lactation period, Barki ewes’ serum albumin and globulin levels significantly increased throughout the late pregnant stage. These results were in line with those of [39] while disagree with those of [51] in camels and [52] in goats. The significant increase in serum albuminn in late pregnancy stage indicating a higher energy need for fetal growth [33]. Meanwhile, the late pregnancy and formation of colostrum are associated with rapid extraction of immunoglobulin from plasma, which may lead to lower level of serum albumin at early lactation [34]. The significant increase in serum globulin in late pregnancy stagemay be due to protein accumulation, particularly immunoglobulins (Ig) into colostrum following lambing [34, 35].

At the late pregnancy, ewes exhibited a marked elevation in serum urea level. This is consistent with that observed in sheep [24] and buffaloes [36]. In contrast [52], reported that there was no discernible variation in the level of urea in goats under various physiological conditions [54]. reported a substantial increase in the serum urea level at early lactating women. The significant increase in serum urea level during late pregnancy may be due to the high energy and protein metabolism required during pregnancy. Alternatively, it could be a consequence of muscle protein being catabolized when significant body reserves are used up [28].

The levels of serum creatinine and total bilirubin during the studied physiological stages did not exhibit any significant differences in Barki ewes. These results were similar to those reported by [37, 38] in goats [24], in sheep, and [39] in cattle. On the contrary [17], observed a significant increase in serum creatinine level in late pregnant goats and camels.

The current study found that the levels of alkaline phosphatase (ALP) activity were significantly higher during the pre-pregnancy stage. These findings are in agreement with those of [52] in goats. In contrast [54], found a considerable increase in ALP activity at early lactation does as compared with other physiological states. It was also reported that the hepatic metabolism may change according the physiological changes, which may also be linked to a decrease in the intake of dry matter around parturition [56]. The physiological role of this enzyme in sheep was associated with early milk production and fetal growth, which may explain the higher activity of ALP in late pregnancy than in early lactation.

The significant increase of serum level of BHBA in late-pregnant and early-lactating ewes is in agreement with the findings of [40] in sheep. However, it differs from the results of [39] who found significant increase of serum BHBA level in the non-pregnant cattle.

In the present study, early lactation sheep had a marked increase in serum NEFA concentrations compared to later physiological phases. These results were in line with those of [39] in cattle, but differ than those of [40] in sheep, who revealed non-significant differences in serum NEFA levels across physiological states. The higher serum NEFA concentrations in early lactating ewes indicate their ability to maximize milk synthesis while consuming less glucose. Additionally, the high levels of growth hormone and low levels of insulin in the blood during this time promote significant mobilization from adipose tissues, supported by the higher serum NEFA concentrations [57].

Thyroid hormone levels were significantly higher in early lactation sheep in the current investigation compared to other physiological stages. Our findings were consistent with those reported in sheep [50], goats [39, 46], and cattle [59]. In contrast, high thyroid activity and circulating TSH concentrations were reported by other authors throughout the pregnancy and postpartum stages in sheep [36] and goats [60]. The effects of galactopoietics, which regulate lactation and stimulate the basic metabolic rate through the metabolism of carbohydrates, lipids, and proteins, may account for the increases in thyroid hormones during early lactation [34].

In the current study, ewes exhibited the highest level of IGF-1 during late pregnancy compared to pre-pregnancy and early lactation stages. These findings resembled those in Gray Shirazi ewes reported by [40]. However, they differed slightly from those reported in another sheep breed [41]. The insulin-like growth factor-1 (IGF-1) hormone, produced by various organs, plays a crucial role in numerous biological processes through both endocrine and autocrine mechanisms, particularly contributing to fetal development [59].

In the current investigation, there was statistically significant increase in MDA along with a substantial decrease in GSH, GPX, CAT and SOD in both late pregnancy and early lactation compared to pe pregnant stage. These results **align** with those reported by [17] in camels [54], in goats, [61] in cattle and [62] in sheep. The current study revealed that in Barki ewes, both late pregnancy and early lactation were associated with the presence of free radicals and oxidative stress. These findings can be attributed to physiological oxidative stress during these periods, characterized by significant energy deficits and substantial metabolic changes, particularly in lipid and protein metabolism. Consequently, the antioxidants required by endogenous antioxidant enzymes to counteract ROS were depleted due to the simultaneous increase in ROS generation during these phases. Additionally, a decrease in daily feed consumption could contribute to a reduction in the activities of antioxidant enzymes, leading to an elevation in the levels of lipoperoxidation products [60].

Comparing the early lactation stage to other physiological stages, the current research found a considerable increase in serum levels of Hp. Consistently, high Hp levels have been documented during the initial stages of lactation in high-producing dairy cows [14, 58] and goats [12]. During early lactation in livestock, the occurrence of negative energy balance (NEB) is common due to the imbalance between energy intake from food consumption and the energy required for milk production. This imbalance may result in an increase in serum levels of Hp during the early lactation stage of negative energy balance [58].

There was not any significant changes of SAA and Fb concentrations among the studied stages. These results were similar to those reported by [12] in Saanen goats. However, other studies reported significant variations in SAA levels during different physiological states in livestock [14,58,59,61] and inflammatory conditions during labor [62,63]. Discrepancies between the results of these studies may be attributed to various environmental factors, including management practices, diet, season, breeding time [64], age, parity, pregnancy status, and milk productivity [14].

In our study, ewes during late pregnancy exhibited significantly lower expression levels of cytokines (*IL-4, IL-6, IL-8*, and *NFKB*) compared to pre-pregnancy and early lactation stages. While similar studies in sheep are scarce for direct comparison with our results [42], investigated the immune gene expression profiles in Holstein cattle during the transition period. They reported up-regulation in the expression levels of certain immunity-related genes, such as TLR 4, 6/7, and β-defensin 5, at the third week post-calving compared to lower expression levels at earlier periods. Additionally, the immunosuppressive *TLR2* gene showed up-regulation during calving and the first week after parturition, followed by down-regulation during the second and third week. Moreover [43], found a significant positive correlation between the expression levels of BDEF5 and post-partum endometrial TLRs genes and the time needed for uterine involution in Holstein cows. In dairy cows [44], reported a significant reduction in the expression level of the *TLR4* gene between **days 7 and 14 after** calving, accompanied by fluctuations in polymorphonuclear neutrophil (PMN) count, which peaked at day 0, declined dramatically at day 7, and increased steadily between days 7 and 21 after calving.

It is worth to mention that the cytokines such as *NFKB, IL-6, IL-8*, and *IL-4* function are **useful indirect indicators of inflammatory process** [45]. The down-regulation of cytokines in late pregnancy among investigate Barki ewes may be attributed to the immunosuppressive nature of the period from parturition to lactation, characterized by impaired neutrophil and lymphocyte functions [46]. Generally, these malfunctioning processes could be linked to an increase in immunological tolerance or a decrease in pro-inflammatory cytokines [47].

The expression profile of antioxidant genes (*SOD, CAT, GPX*, and *Nrf2*) were significantly increased in the pre-pregnan Barki ewes compared to late pregnancy and early lactation stages. To our knowelege, there is a limited information on ovine gene expression profile of antioxidant markers during different physiological condtions. However, in a study on dromedary camels during the periparturient period [48], investigated the expression profile of antioxidant-related genes. The authors found that *SOD1, SOD3, CAT*, and *GPX* genes exhibited upregulation at both (-14 day) prepartum and (+ 14 day) postpartum compared to their values at calving. Additionally, there was a significant overexpression of stress-induced phosphoprotein (STIP1), stress-associated endoplasmic reticulum proteins (SERP2), and oxidative stress-responsive kinase 1 (OXSR1) at (-14 day) prepartum compared to their comparable values at calving and (+ 14 day) postpartum.

Antioxidants function as scavengers or detoxifiers of ROSs by inhibiting their generation, which is the source of free radicals [49]. Consequently, antioxidant defense mechanisms, such as endogenous *GPx, CAT*, and *SOD* [50] may be activated. However, the primary defense against oxidative stress involves controlling the expression of cytoprotective genes through the Nrf2 stress response pathway [51]. The alteration in the expression pattern of antioxidant markers in tis investigation could be attributed to the high energy and oxygen requirements during pregnancy, which may impact the expression level of the antioxidant markers [52]. Moreover, the high molecular oxygen levels needed for aerobic metabolism to support extensive milk production for newborn feeding may contribute to increased reactive oxygen species (ROS) production [53, 54]. Additionally, lipid peroxidation following calving was reported to increase as well [15]. The metabolic challenges in the ewe’s body during the postpartum period may result in a negative energy balance [53], leading to an increase in the production of reactive oxygen metabolites (ROMs) [54]. This significant alteration in the energy, minerals and vitamins requirements during the late pregnancy may induce a pro-oxidant shift in the redox balance, thereby affecting the oxidative stress markers [55].

**This is the first study to correlate the gene expression and metabolic profile of cytokines and antioxidant markers in Barki ewes during different physiological satges**. According to our findings some parameters were correlated as follows; the serum levels of SAA were positively correlated with mRNA levels of IL4 (*r* = 1 and *p* = 0.009) in non-pregnant non lactating group. The serum levels of Hp were positively correlated with mRNA levels of GPX and Nrf2 gene (*r* = 0.999 and *p* = 0.02, *r* = 1and *p* = 0.011, respectively) in late pregnant group. The serum levels of GPX were positively correlated with mRNA levels of CAT and GPX gene (*r* = 1 and *p* = 0.01, *r* = 0.997 and *p* = 0.04, respectively), serum levels of Hp were negatively correlated with mRNA levels of CAT (*r*= -1 and *p* = 0.01), serum levels of SAA were negatively correlated with mRNA levels of SOD gene (*r*= -0.998 and *p* = 0.04) and serum levels of Fb were positively correlated with mRNA levels of IL4 gene (*r* = 1 and *p* = 0.01) in early lactating group.

**The present study showed some limitations**, which should be considered in future studies. Firstly, the current investigation was carried out on a limited number of Barki ewes. As a result, more research on a large number of ewes is needed. Secondly, this investigation should be applied on different breeds of sheep for more an accurate health judgment. Thirdly, a limited number of genes related to immunity and antioxidant were examined. Thus, a wide range of factors has to be taken into account in subsequent research.

## Conclusion

The results of our investigation concluded that across several physiological stages in Barki ewes, there were significant differences in a variety of metabolic profiles as well as changes in the gene expression profiles of cytokines (IL-4, IL-6, IL-8, and NFKB) and antioxidants (SOD, CAT, GPX, and Nrf2) markers.

Furthermore, identifying differences in these genes’ levels of expression improves our comprehension of the genetic foundation of the adaptation process. These results, which may represent physiological variations, might be used to predict the animal’s susebtability or resistance to stress under different physiological conditions. Moreover, these findings may have implications for the breeding strategy and selection processes used for Barki sheep raised under harsh environmental conditions.

## Data Availability

No datasets were generated or analysed during the current study.
